# Study on Microstructure and Property Regulation of 18Ni350 Maraging Steel Fabricated by Selective Laser Melting and Its Corrosion Resistance to Molten Aluminum

**DOI:** 10.3390/ma19143030

**Published:** 2026-07-14

**Authors:** Lei Zhang, Luwei Zeng, Zhong Zeng, Jiuzhang Li, Yanghui Jiang, Bing Yang

**Affiliations:** School of Power & Mechanical Engineering, Wuhan University, Wuhan 430072, China; leopold@whu.edu.cn (L.Z.); 15329303240@163.com (L.Z.); 2019302080265@whu.edu.cn (Z.Z.); 2024282080078@whu.edu.cn (J.L.);

**Keywords:** selective laser melting, 18Ni350 maraging steel, Electro-Spark Deposition, TiB_2_, molten aluminum corrosion resistance

## Abstract

**Highlights:**

This study aims to investigate the influence mechanism of heat treatment on the microstructure and mechanical properties of 18Ni350 maraging steel, as well as the improvement effect and failure mechanism against molten aluminum corrosion of 18Ni350 maraging steel via electrical discharge-deposited TiB_2_ coating on its surface.

- After solution treatment at 850 °C for 1 h followed by aging at 520 °C for 6 h, the specimen obtained an ultimate tensile strength of 2476 MPa, a yield strength of 2408 MPa, and an elongation after fracture of 4.6%.
- We elucidated the mechanisms of microstructure evolution induced by different heat treatment processes.
- We revealed the distinct failure mechanisms of 18Ni350 steel versus TiB_2_ coating under 750 °C molten aluminum corrosion.

**Abstract:**

The influence of different heat treatment processes on the microstructure and mechanical properties of 18Ni350 maraging steel manufactured by selective laser melting and the corrosion resistance of TiB_2_ ceramic coatings Electro-Spark-Deposited on its surface when immersed in high-temperature molten aluminum have been investigated in the present study. The microstructures and mechanical properties of the differently heat-treated samples were analyzed using various precision instruments. The results reveal that the as-built sample exhibits a microstructure composed of cellular and columnar dendritic grains. After solution treatment, the microstructure fully transforms into lath-like martensite. After direct aging treatment, the cellular structures diminish, while precipitates at grain boundaries proliferate with increasing aging temperature. SAT- treatment achieves full microstructural homogenization, featuring fine-lath martensite and a small amount of randomly distributed austenite particles. DA- and SAT- significantly improve the strength, hardness and modulus of samples and were found to reduce the toughness and plasticity. After solution treatment at 800 °C for 1 h followed by aging treatment at 520 °C for 6 h (SAT 800-520), the specimen achieved an UTS of 2476 MPa while maintaining an EL of 4.6%. The TiB_2_ coating and the Cr interlayer deposited via ESD form a continuous interfacial bond with the substrate, demonstrating favorable adhesion. After 4 h of static immersion in high-temperature molten aluminum, the coating remains intact without complete delamination, delivering effective protection to the substrate.

## 1. Introduction

Selective Laser Melting (SLM) represents a cutting-edge additive manufacturing technology that stands out from conventional subtractive methods [1]. The SLM technology revolutionized the creation of mold conformal cooling water channels, offering innovative opportunities that transcend conventional limitations. By eliminating design constraints inherent in traditional drilling methods, it circumvented subsequent manufacturing challenges associated with channel processing. Simultaneously, this approach significantly enhances the cooling efficiency of molds and accelerates the overall manufacturing process.

Owing to its exceptional mechanical properties, including extraordinary strength and ductility, maraging steel finds extensive application across various industries such as mold production, precision tools, aerospace, and other related sectors [2]. The SLM technique has emerged as a significant production method for maraging steel, with the 18Ni350 grade successfully fabricated and extensively studied. In their work, Duan et al. [3] examined the impact of direct aging (DA) heat treatment on the microstructure and mechanical behavior of additively produced 18Ni350 maraging steel. Their findings revealed that aging significantly enhances the material’s mechanical performance. Li et al. [4] studied the influence of interlayer temperature on the microstructure and mechanical properties of wire arc additively manufactured 18Ni350 maraging steel. It was discovered that modulating the interlayer temperature near the martensite start point effectively enhances the microstructure and achieves an optimal balance between strength and ductility in maraging steel fabricated via DED.

Despite the growing number of activities in this research field, investigations into metal powder raw materials and SLM-based manufacturing processes remain scarce. Casalino G et al. [5,6] employed a combined approach of experimentation and continuous statistical optimization to investigate the SLM fabrication of 18Ni300 maraging steel. Their findings revealed that material hardness, strength, and surface roughness increase with sample density. Afkhami et al. [7] investigated the influence of laser power, scanning speed, hatch spacing, and layer thickness on critical attributes of SLM-processed 18Ni300 maraging steel, including molten pool morphology, surface quality, and internal porosity. Their study revealed the effects of these parameters on both external and internal microstructural characteristics. Several key parameters significantly influence the melt pool characteristics and surface integrity. These factors encompass recoil pressure, insufficient orbital overlaps, interlayer re-melting, Plateau–Rayleigh instability, and material aggregation resulting from the Marangoni effect. These studies serve as valuable references for optimizing process parameters and analyzing properties of 18Ni350 maraging steel produced via SLM, thereby offering a robust experimental foundation for advancing SLM applications in mold manufacturing.

At the same time, adequate heat treatment plays a pivotal role in enhancing product performance during industrial production. The 18Ni350 maraging steel undergoes an aging process, which enables remarkable enhancements in material characteristics. Within the SLM forming procedure, a rapid cooling of the powder following laser melting may lead to enhanced precipitation within the sample structure. Additionally, heat treatment steps may influence both the creation and spatial arrangement of precipitated phases. Consequently, this can have a direct impact on the overall performance and properties of the final sample [8]. Furthermore, the SLM forming process exposes the sample to cyclic cooling and thermal fluctuations, leading to a significant temperature gradient in the formed layer. Resulting from this temperature gradient is substantial internal stress within the layer, which induces deformation and crack propagation. Understanding and addressing these stress-related issues are critical for advancing SLM technology. Fortunately, appropriate heat treatment strategies can mitigate these thermal stresses in the as-built component. Consequently, investigating the heat treatment process during the SLM fabrication of 18Ni350 stainless steel yields valuable insights that can enhance its mechanical properties and overall performance. While Duan et al. [3] have examined the influence of the direct aging process on the microstructure and mechanical properties of 18Ni350 maraging steel produced via SLM, their findings lack comprehensive analysis and scope. In consequence, there remains an urgent need for more comprehensive and methodical research into this area. Such studies should involve detailed characterization of its microstructure and mechanical properties across scales, as well as other critical aspects influencing performance.

The corrosion failure of die-casting molds caused by molten aluminum at high temperatures plays a significant role in limiting their service life [9,10]. Applying corrosion-resistant coatings to mold surfaces aims to enhance their durability. Electro-Spark Deposition (ESD), a surface coating method, employs high-current pulses to apply material layers onto metallic substrates. This technique is widely used for on-site component repair in various sectors, including aerospace, mold production, and cutting tool manufacturing, as well as the development of wear-resistant, corrosion-resistant, and heat-resistant coatings [11,12].

TiB_2_ is widely applied to cemented carbide cutting tools as a coating material, thanks to its superior hardness and high melting point. Kou et al. [13] developed TiB_2_ coatings on cemented carbides using electrophoretic deposition in molten salts, which exhibited high hardness and strong adhesion properties, along with a reduced friction coefficient and minimized wear rate. Additionally, Luo et al. [14] utilized Electro-Spark Deposition (ESD) to deposit TiB_2_ coatings and examined their microstructure, phase composition, hardness, and interfacial element distribution. The findings demonstrated significant elemental diffusion at the interface, indicating strong metallurgical adhesion between the coating and substrate. Despite advancements in this research area, investigations into the corrosion behavior of TiB_2_ coatings deposited via ESD in high-temperature molten aluminum remain limited. Accordingly, assessing the corrosion resistance of TiB_2_ coatings fabricated using ESD on SLM-processed 18Ni350 substrates under molten Al environments holds significant importance in prolonging mold lifespan within foundry applications.

## 2. Materials and Methods

**Figure 1:**
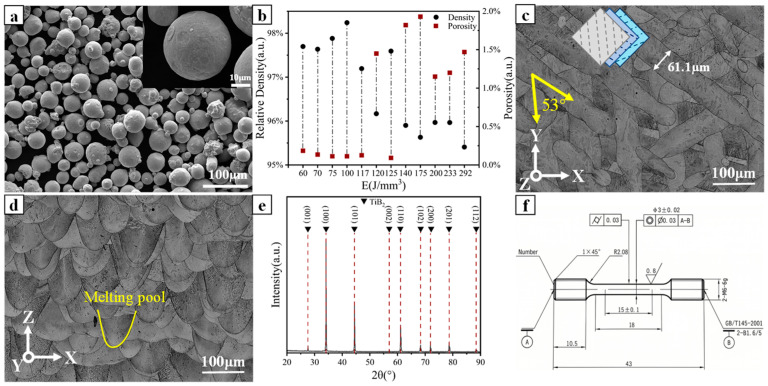
Material preparation and preliminary tests: (**a**) Morphology of the powder of 18Ni350 steel, (**b**) relative density and porosity, (**c**,**d**) morphology of horizontal and vertical section of as-built sample, respectively, (**e**) XRD spectrum of TiB_2_ electrode, (**f**) dimension of tensile specimen.

The morphology of the 18Ni350 powder (provided by Jiangsu Vilory Advanced Materials Co., Ltd., Xuzhou, China) was observed under a SEM (MIRA 3) equipped with an X-Max 20 spectrometer (Aztec Energy, Wauchula, FL, USA), with an acceleration voltage of 20 kV and shown in Figure 1a. A SLM device (BLT-A160, Xi'an Bright Laser Technologies Co., Ltd., Shenzhen, China) was used to fabricate the experimental parts in argon protective atmosphere. The main chemical composition of powder provided by Jiangsu Vilory Advanced Materials Co., Ltd. is listed in Table 1. Different process parameter sets, including scanning speed (v), powder layer thickness (h), laser spot diameter (d), laser power (P) and energy density (E) were designed, as shown in Table 2. The porosity measured from surface morphology images and the relative density determined by the Archimedes drainage method (LSD-1200F, Xiamen Laiside Scientific Instrument Co., Ltd., Xiamen, China) are shown in Figure 1b. Specimen No. 1 presents the highest relative density and the lowest porosity, and its corresponding forming parameters are thus determined as the optimal fabrication parameters. Optimized process parameters are as follows: laser power of 300 W, scanning rate of 1000 mm/s, layer thickness of 40 μm and spot diameter of 30 μm. The corresponding volume energy density is 100 J/mm^3^. An inter-planar rotation angle of 53° was adopted. Figure 1c shows the scanning traces revealed in horizontal section of parts, and Figure 1d reveals the partial overlaps between hatches in the vertical section which is observed under a SEM (MIRA 3). Cylindrical Cr and TiB_2_ rods with Φ3 mm × 30 mm were used as electrodes for ESD. The XRD diffraction pattern of the TiB_2_ electrode is shown in Figure 1e. The substrates used for ESD were the 18Ni350 maraging steel specimens with optimal comprehensive mechanical properties after heat treatment (SAT 800-520). The longitudinal sections of the substrates were polished and cleaned prior to deposition. The dimensions of the tensile specimen are shown in Figure 1f.

After fabrication, based on the phase transformation rules of similar maraging steels [15,16,17,18,19] and hardening curves reported [20], considering the heating rate of the muffle furnace (XMT-800, Shanghai Gongbao Electric Co., Ltd., Shanghai, China), 9 heat treatment processes were set. These can be categorized into three main types: solution treatment (ST), direct aging treatment (DA), and solution-aging treatment (SAT). Samples are named differently based on their heat treatment temperature. The specific heat treatment processes and corresponding sample names are listed in Table 3.

Nital solution of 4% was used to reveal the microstructure. The microstructure, micro-area composition and electron backscatter diffraction (EBSD) were observed under a SEM (MIRA 3) equipped with an X-Max 20 spectrometer (Aztec Energy, Wauchula, FL, USA), with an acceleration voltage of 20 kV. Analysis featured an EBSD step size of 0.2 μm, analyzed area of 276.7 × 208.0 μm^2^, phase-indexing settings for the Iron bcc and Iron bcc (old), and the post-processing cleanup procedure using Aztec Crystal 2.1 software. According to ASTM E8 standards, the tensile specimens were tested at a displacement of 1.5 mm/min with an electro-hydraulic servo mechanical testing machine (MTS Landmark 370.02, MTS Systems, Beijing, China). In this study, the mechanical properties under each heat treatment condition were tested along the longitudinal section direction for 3 times to guarantee the repeatability of data. After polishing, samples were tested using a micro Vickers hardness tester (Micro Vicker HV-1000 A, Sinowon Innovation Metrology Manufacture Limited, Shanghai, China) under a load of 500 gf and a dwell time of 10 s, with the average value calculated from ten tests after discarding the maximum and minimum values to ensure data reproducibility.

An ESD device (ESD-150, ITECH Electronics (Nanjing) Co., Ltd., Nanjing, China) was used to deposit TiB_2_ coating, with pure Ar gas (99.8%) as the shielding gas. Prior to deposition, the substrate specimens were machined into 10 mm × 10 mm × 10 mm blocks using a wire electrical discharge machining (WEDM) system. The surfaces were sequentially ground with 800#, 1200# and 2000# SiC sandpapers to a mirror finish, followed by ultrasonic cleaning in acetone for 15 min to completely remove surface oil and machining residues. The deposition parameters were: deposition voltage 100 V and deposition capacitance 20 μF, 40 μF, 60 μF, 80 μF, 100 μF, and 120 μF. The Cr transition layer and the TiB_2_ coating were deposited under identical deposition parameters. Specifically, the Cr transition layer was uniformly deposited with 2 layers, whereas the TiB_2_ coating was deposited with 4 layers. Phase composition of the coatings was measured with an X-ray diffractometer (Rigaku Smartlab SE, Tokyo, Japan). The Kα radiation of Cu served as the X-ray source (λ = 1.5418 Å). A scanning rate of 5°/min and a scanning range of 20–90° were adopted. The wear resistance of the coating was tested using an MS-T3001 (Jinan Winner Particle Instrument Stock Co., Ltd., Jinan, China) friction and wear tester, with a rotational speed of 200 r/min, a normal load of 5 N, a test duration of 10 min and a rotation radius of 3 mm. A muffle furnace (XMT-800, Shanghai, China) was used to heat a pure Al block to 750 °C. Aluminum blocks (99.99 wt.%) were placed in a magnesia cupel and heated in a furnace. After the aluminum was fully molten, the specimens (10 mm × 10 mm × 10 mm) were immersed into the molten aluminum of 5 mm and held isothermally for 30–240 min. Upon completion of the holding period, the specimens were removed from the melt and furnace-cooled.

## 3. Results

### 3.1. Microstructure Characterization of As-Built Sample

**Figure 2:**
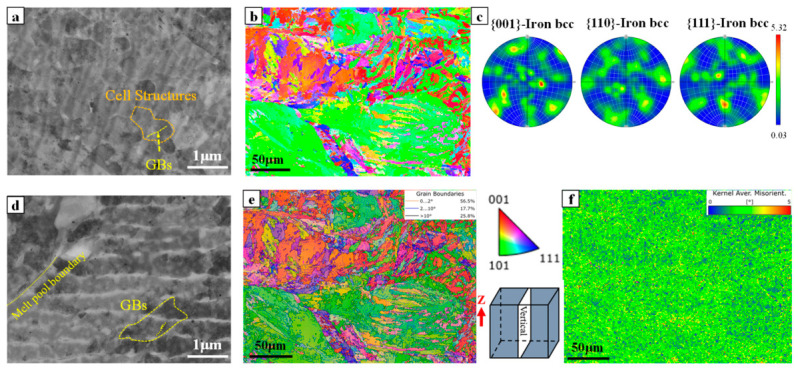
SEM morphology and EBSD analysis of as-built sample: (**a**,**d**) BSE images, (**b**) IPF, (**c**) PFs, (**e**) GBs, (**f**) KAM.

The initial microstructure consists of equiaxial or columnar cellular structures with non-uneven sizes, as shown in Figure 2a,d, which is consistent with the work of Mei et al. [21]. Martensite grain boundaries (GBs) were observed traversing near the cell walls or through the interior of the cellular structures, marked by yellow dashed lines in the figure. An important distinction between martensite grain boundaries and cell walls is their impact on the matrix integrity. Cell walls do not split the matrix into many grains like martensite grain boundaries do. This is because cell walls consist of entangled dislocations in nature but do not create a general sharp interface.

The IPF map of the vertical section of the as-built sample is shown in Figure 2b. A relatively chaotic color distribution can be observed, with regions exhibiting a (101) preferred orientation within the melt pool. This may be attributed to the extremely high temperature gradient (10^4^ K/m < G < 10^5^ K/m) within the melt pool during the SLM process, where the microstructure grows inward from the melt pool boundary, forming the characteristic of (101) preferred growth. From Figure 2c it can be observed that the color distribution is relatively chaotic, and the maximum pole density is less than 5.32, indicating that the grains did not preferentially grow along a specific crystallographic direction and the texture is very weak. This may be related to the alternating layer rotation scanning strategy in SLM, which may cause alternating changes in the direction of heat flow for each layer. Figure 2e shows the grain boundary distribution map, where the proportions of low-angle grain boundaries (LAGBs) and high-angle grain boundaries (HAGBs) are 74.2% and 25.8%, respectively. Sub-grain boundaries account for 56.5% of the LAGBs. Generally, HAGBs possess higher binding energy and contribute more to grain boundary energy, thus forming at the boundaries of martensite structures. LAGBs have lower binding energy and lower grain boundary energy, thus forming within the crystal. Within LAGBs, dislocations can easily arrange along the grain boundaries, forming aligned dislocations. The kernel average misorientation (KAM) map is shown in Figure 2f. As illustrated in the KAM map, the specimen exhibits small local misorientation, slight lattice distortion, and a low level of microscopic residual stress.

### 3.2. Microstructure Characterization of Solution Treatment Samples

**Figure 3:**
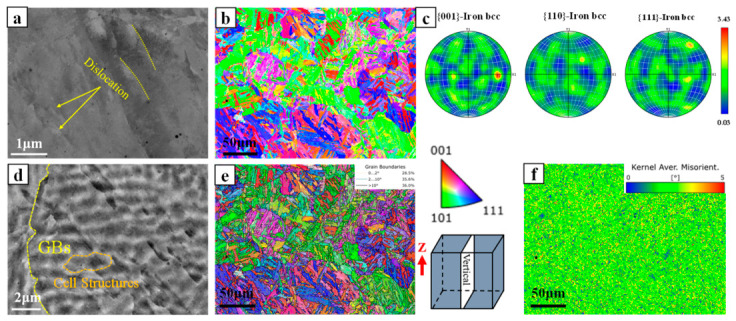
SEM morphology and EBSD analysis of ST 800 and ST 850 samples: (**a**) BSE images of ST 800, (**b**) IPF of ST 800, (**c**) PFs of ST 800, (**d**) BSE images of ST 850, (**e**) GBs of ST 800, (**f**) KAM of ST 800.

After solution treatment, the sample microstructure transformed into dislocation martensite. Dislocations distributed within the martensite can be clearly observed in the ST 800 sample, as shown in Figure 3a. Figure 3b shows that with increasing the solution temperature to 850 °C, the martensite size increased, and the grain boundaries became larger. Cell structures identical to those in the as-built state were observed within the martensite laths. He et al. [22] found in their study that the austenite start transformation temperature (A_s_) for 18Ni350 maraging steel is around 700 °C. When the temperature rises to about 790 °C, the transformation to austenite is complete (Af). Upon rapid cooling, austenite rapidly transforms to martensite around 170 °C, and the martensitic transformation finishes around 50 °C. During rapid cooling, austenite quickly transforms to martensite. Due to the excessively high cooling rate (>1000 K/min), it is difficult for austenite to completely transform into lath martensite, which may result in the small amount of cellular structure observed in the ST 850 specimen.

EBSD analysis of the ST 800 sample is shown in Figure 3b–f. From the IPF map, it can be seen that after solution treatment, the sample’s orientation appears more chaotic. From the PF maps, it is found that the maximum pole density is less than 3.43, indicating a very weak texture in the surface sample with no obvious preferred orientation. From Figure 3e, it is observed that the HAGB content increased to 36%, while the sub-grain boundary content decreased to 28.5%. This is because solution treatment dissolves solute atoms into the grain interior, eliminating local supersaturation near grain boundaries [8]. Due to the short holding time, LAGBs with lower energy and dislocation walls are eliminated more quickly, leading to the phenomenon of higher HAGB content. The KAM map may indicate that after solution treatment, the residual stress on the sample surface further decreased.

### 3.3. Microstructure Characterization of DA Samples

**Figure 4:**
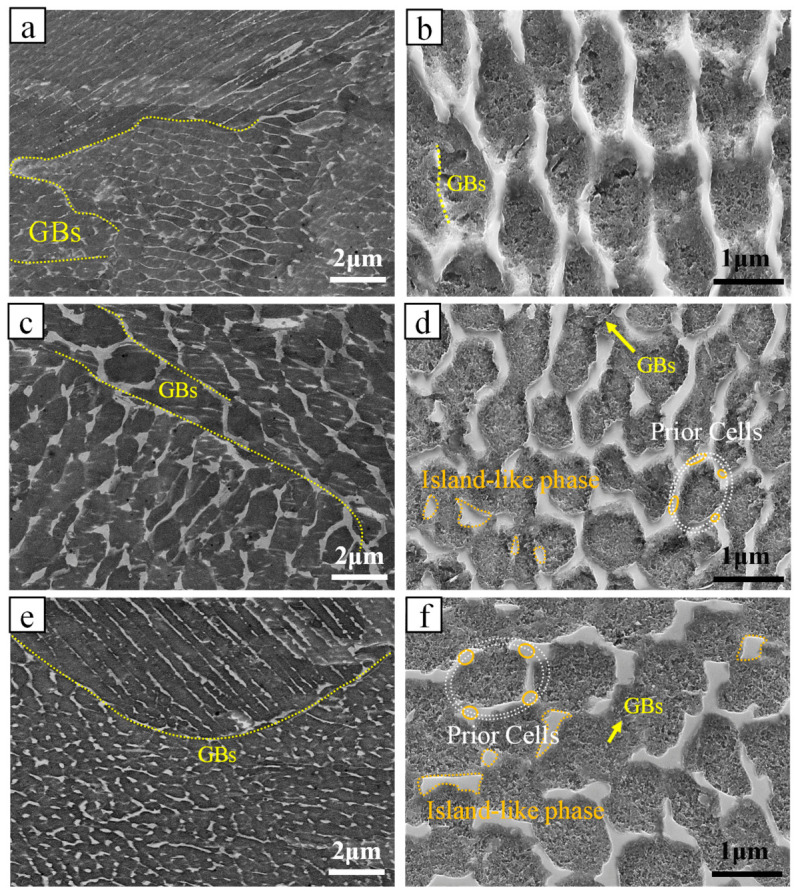
SEM morphology of DA samples: (**a**,**b**) DA 500, (**c**,**d**) DA 520, (**e**,**f**) DA 540.

After aging at 500 °C, the cell walls exhibited a certain degree of deformation, which was particularly pronounced near the martensite grain boundaries, as shown in Figure 4a. In this state, the cell walls remained closed. Figure 4b shows martensite GBs passing through the interior of the cell walls, and the cell walls do not split the matrix like martensite GBs (sharp boundaries) do. Figure 4c clearly shows martensite GBs passing through or near the cell walls. Influenced by the martensite GBs, the deformation of the cellular structure is more evident. Figure 4d shows that after aging at 520 °C, partial breakage of the cell walls occurred, and a small number of island-like morphologies began to appear. After aging at 540 °C, the deformation of cell walls became more pronounced. In areas traversed by martensite grain boundaries, a large number of cells fractured and disintegrated, as shown in Figure 4e. From Figure 4f, it can be seen that after aging at 540 °C, cell walls extensively disintegrated, and numerous discrete island-like morphologies appeared, which is a typical over-aged morphology [21]. The decomposition of cell walls is achieved through dislocation migration and natural dissipation (i.e., the recovery process), driven by the energy stored by dislocations. It has been reported [20] that 18Ni350 nearly reaches its peak strength after aging at 500 °C for 3–4 h, and the time required to reach peak strength becomes shorter at higher aging temperatures. However, the cellular structure of SLM-fabricated 18Ni350 remained well-defined even after aging at 500 °C for 6 h, and a significant amount of cell wall structure was still retained after aging at 540 °C for 6 h. The cellular structure of SLM 18Ni350 steel may possess higher thermal stability than typical cellular structures formed by severe deformation; this may due to certain special dislocation configurations having lower stored energy [23].

### 3.4. Microstructure Characterization of SAT Samples

**Figure 5:**
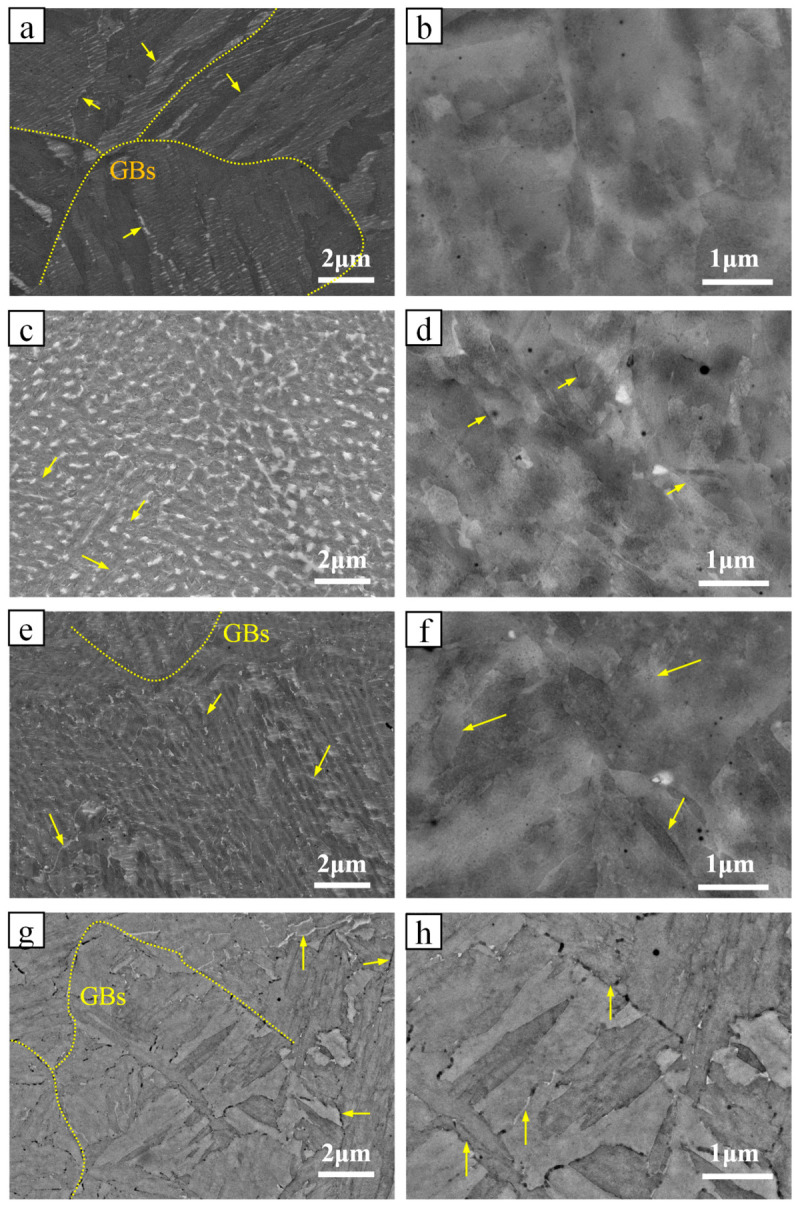
SEM morphology of SAT samples: (**a**,**b**) SAT 800-500, (**c**,**d**) SAT 800-520, (**e**,**f**) SAT 800-540, (**g**,**h**) SAT 850-520.

Figure 5 shows the morphology of SLM 18Ni350 maraging steel after different solution and aging treatments. It can be seen that the boundaries of the laser melt pools have completely disappeared. During the solution treatment process, the specimen is heated to the high-temperature single-phase region, where all phases are completely dissolved, eliminating the laser melt pools. The residual stress generated during the specimen forming process is reduced or even eliminated. Meanwhile, the formation of this supersaturated solid solution prepares for the subsequent aging heat treatment and the precipitation of intermetallic compounds [24].

In Figure 5a, the martensite GBs dividing the matrix, the columnar structures distributed within the grain boundaries are clearly observed. The martensite GBs are marked by yellow arrows and dashed lines in the figure. Intermetallic compounds are dispersed at the martensite grain boundaries and dislocations, as shown in Figure 5b. Figure 5c shows that after solution + aging treatment, the cell walls are almost completely destroyed, presenting a chaotically distributed island-like structure. Figure 5d indicates that the microstructure still consists of tangled dislocations. In Figure 5e, the martensite GBs dividing the matrix and the almost completely decomposed cellular structure are still visible. The cell walls have nearly completely decomposed, and few island-like structures remain, indicating that the 800 °C solution and 540 °C aging heat treatment has completely decomposed the dislocations in the cell walls, causing the cell walls to disappear. In Figure 5f, dislocations could still be seen, and precipitated phases are dispersed around the martensite grain boundaries. After SAT 850-520 treatment, no cellular structure is observed on the specimen surface; instead, large martensite laths are present, and the martensite grain boundaries are clearly visible, as shown in Figure 5g.

### 3.5. Mechanical Properties

**Figure 6:**
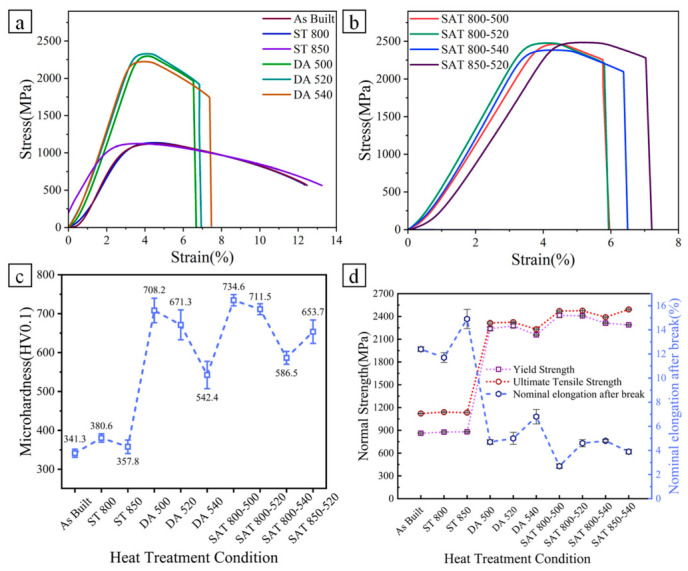
Measurement results of tensile properties and microhardness: (**a**,**b**) Strain–stress curves, (**c**) variation in microhardness with heat treatment conditions, (**d**) variation in tensile properties with heat treatment conditions.

The variation trends of tensile curves and tensile properties are shown in Figure 6a,b and d, respectively, with specific values summarized in Table 4. Each condition was tested three times to obtain the average and standard deviation.

As shown in Figure 6 and Table 4, aging treatment effectively improves the mechanical properties of 18Ni350 steel. Solution plus aging treatment further increases the strength and hardness of the specimens, and the strength exceeds the strength of conventional 18Ni350 steel (2.4 GPa) [25,26]. Among them, the SAT 800-520 specimen exhibited the optimal comprehensive mechanical properties, with an UTS of 2476 MPa, a YS of 2414 MPa, and an EL of 4.6%. The ductility decreases with the increase in specimen strength, which arises from the trade-off between inhibiting dislocation motion and enhancing dislocation mobility. It is difficult to simultaneously improve both properties in most deformation models dominated by dislocation slip [27]. UTS and EL exhibit opposite variation trends with rising aging temperature, which may be related to the disintegration of cellular structures and the increased austenite content at higher aging temperatures.

The variation trend of microhardness is consistent with that of strength. After solution treatment at 800 °C, the microhardness of the specimens increases slightly. Further raising the solution temperature leads to a reduction in microhardness, which can be attributed to the growth of recrystallized grain size [28]. Microhardness rises remarkably after aging, and a further improvement in microhardness is achieved via combined solution and aging treatment.

### 3.6. Microstructure Characterization of TiB_2_ Coatings

**Figure 7:**
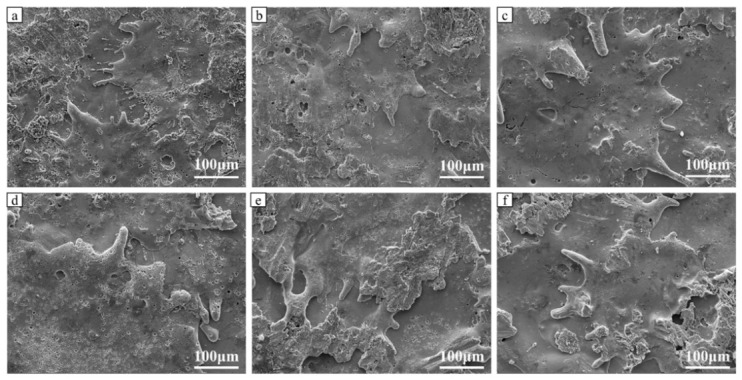
Morphology of TiB_2_ coatings under different conditions: (**a**) 20 μF, (**b**) 40 μF, (**c**) 60 μF, (**d**) 80 μF, (**e**) 100 μF, (**f**) 120 μF.

Figure 7 shows the surface morphology of coatings prepared under 100 V voltage with different capacitance parameters. It can be seen that the coating surface has cloud-like deposition spots, accompanied by some pits and granular splatters, resulting in a relatively high surface roughness. The main reason for splatter generation is that during the deposition process, the discharge from pulses melts the material at the electrode tip, which is then flung out by the high-speed rotating electrode and splattered onto the substrate surface. The ESD process is cumulative of multiple single-pulse discharges. Through multi-point discharges, the deposition spots connect to form a stacked morphology, simultaneously improving surface flatness significantly [29].

Some micro-cracks and tiny pores exist on the coating surface. This may be because during deposition, the melting, vaporization, and solidification of the substrate and electrode materials are completed within an extremely short time (10^−6^–10^−5^ s) [30]. Instantaneous generation of a large amount of heat energy accelerates air flow, affecting the argon protection to some extent. External gases supplement the interior, causing the strengthened area to absorb dissolved gases, thus forming pores. During ESD, the pulse discharge time is very short, and temperature changes rapidly. The coating and substrate undergo volume expansion during rapid heating, generating thermal stress. When stress accumulation exceeds the coating’s own strength limit, the release of thermal stress in the coating produces micro-cracks [31]. Additionally, after high-temperature discharge, substrate materials such as Fe and Cr diffuse into the molten coating. Due to different expansion coefficients, microstructural stress is generated during cooling and may lead to micro-cracks under applied forces [32].

During ESD coating deposition, the relationship between pulse energy and voltage/capacitance is given by the following formula:

(1) $$E=\frac{1}{2}CU^{2}$$

where E is the pulse energy, C is the capacitance during deposition, and U is the voltage during deposition. The formula shows that under constant voltage, increasing capacitance leads to increased pulse energy. From Figure 7, it can be seen that as capacitance increases, the size of the blocky spatters on the coating surface increases. This is because increased capacitance leads to higher pulse energy, larger molten droplet size from electrode melting, and ultimately larger spatter size. Furthermore, as capacitance increases, the number of surface micro-cracks initially increases and then decreases. This may be attributed to the increased deposition efficiency of the coating due to higher pulse energy, and the continuous accumulation of thermal stress within the coating, leading to a significant increase in surface crack count [33]. At higher capacitances, due to increased coating deposition efficiency, defects like pores decrease, effectively reducing residual stress on the coating surface and minimizing crack formation.

**Figure 8:**
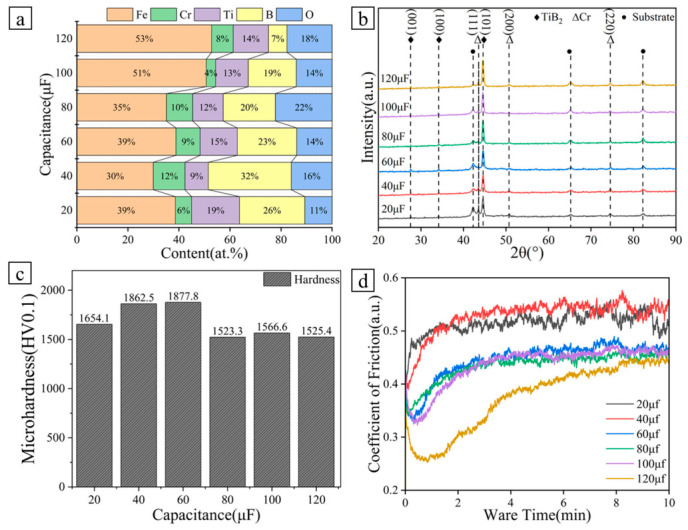
Analysis of coating surface properties in different capacitances: (**a**) Surface element contents, (**b**) XRD patterns, (**c**) microhardness, (**d**) friction curve.

Figure 8a shows the variation in surface elemental composition of the coatings. It can be seen that the coating surface contains a large amount of Fe, and the Fe content increases with increasing capacitance. The atomic diameters of Ti (0.146 nm) and Fe (0.124 nm) are relatively large, resulting in significant diffusion resistance in the solid state and requiring high diffusion activation energy. Long-distance diffusion across the surface coating is difficult to complete within a short time (μs) [33]. This indicates that melting of both electrode and substrate materials occurs during ESD. In the liquid state, rapid element transfer can be achieved through convection and diffusion, allowing Fe from the substrate to enter the coating. As the capacitance increases during preparation, the pulse energy rises, intensifying elemental diffusion. This causes Fe from the substrate to diffuse into the coating, leading to an increase in the Fe content within the coating. Even with argon used as a protective gas during the coating deposition process, O was detected on the coating surface. Furthermore, as the capacitance increases, the oxygen content shows an upward trend. This is because higher capacitance leads to greater energy per unit area and accelerates air flow, which somewhat compromises the protective effect of the argon gas. Consequently, some oxygen becomes involved, causing oxidation on the material surface.

Figure 8b shows the XRD patterns of coatings prepared under different capacitance conditions. It can be seen that the coating primarily consists of TiB_2_ and Cr phases. Intermediate products of TiB_2_ such as TiB, Ti_3_B_4_, and Ti_2_B_5_ were not detected. To a certain extent, this indicates that the ESD technique could mitigate material heterogeneity. Moreover, the TiB_2_ shows a pronounced preferred growth orientation in the (101) direction, which differs from the (100) preferred growth orientation of the electrode material.

Figure 8c shows the variation in microhardness of TiB_2_ under different capacitances. The coating hardness generally exhibits a downward trend as the capacitance increases. This is attributed to the increased pulse energy during deposition at higher capacitance, which enhances the diffusion of Fe from the substrate and Cr from the intermediate layer into the coating, while Ti also diffuses into the intermediate layer and even the substrate. The intensified elemental diffusion leads to the formation of low-hardness components within the coating, resulting in a gradient decrease in coating hardness [34]. This elemental diffusion is also the reason why the coating hardness is much lower than the theoretical hardness of TiB_2_ (greater than 3000 HV).

Figure 8d shows the friction and wear curves of coating surfaces prepared under different capacitance conditions. It can be observed that as the deposition capacitance increases, the friction coefficient displayed an initial increase followed by a subsequent decrease. This is because, under low deposition capacitance conditions, the deposition efficiency increases and defects such as pores decrease, effectively reducing residual stress on the coating surface, improving the deposition morphology, and lowering surface roughness. Among them, the friction coefficient is highest at 40 μF (0.51) and lowest at 120 μF (0.46).

**Figure 9:**
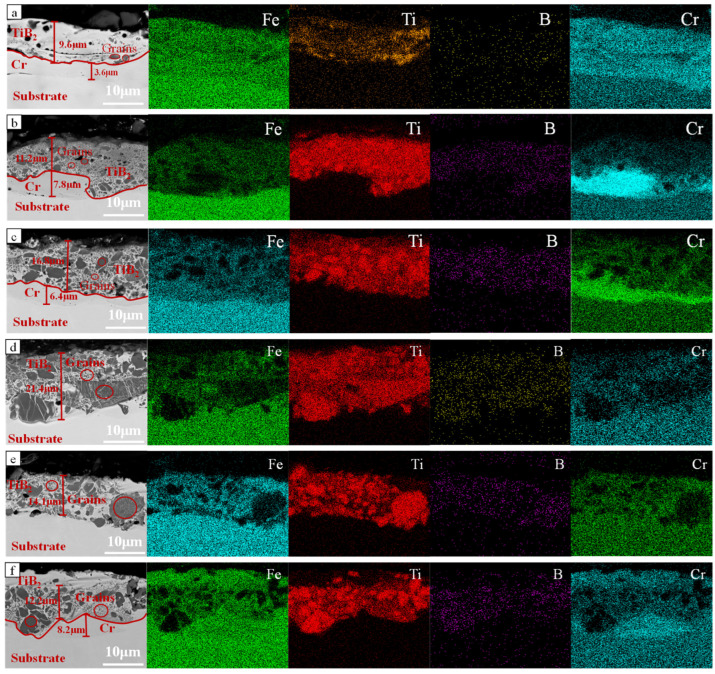
Coating cross-section morphology under different conditions: (**a**) 20 μF, (**b**) 40 μF, (**c**) 60 μF, (**d**) 80 μF, (**e**) 100 μF, (**f**) 120 μF.

Figure 9 shows the cross-sectional morphology and atomic distribution maps of coatings prepared under different capacitance conditions. It can be seen that coatings prepared under 20~60 μF and 120 μF conditions show good bonding between the intermediate layer and the substrate, as well as between the coating and the intermediate layer, with no obvious defects such as voids. Cracks on the coating surface do not penetrate deep into the substrate. In some coatings, a distinct Cr intermediate layer is not visible, primarily due to the good wettability between Cr and Fe [35]. In the molten pool generated by the spark, they can fully contact and flow well, forming a strong metallurgical bond between the substrate and the intermediate layer with a uniform and continuous interface, without a distinct Cr layer appearing. As the capacitance increases, the TiB_2_ crystal size tends to increase, and the coating consists more of large crystal clusters. This is because, with increasing pulse energy, the size of the molten pool formed on the coating surface increases, elemental diffusion intensifies, and the TiB_2_ crystallization rate improves, leading to the formation of larger crystals.

**Figure 10:**
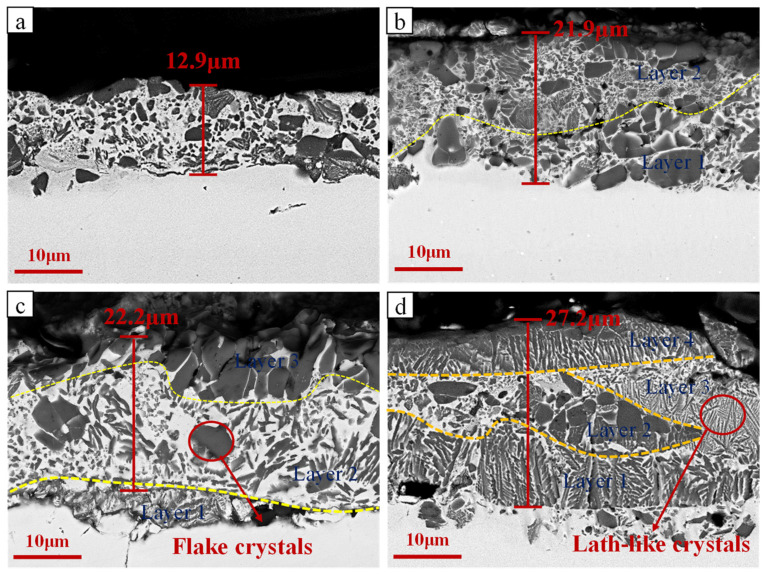
Different layers of TiB_2_ coatings: (**a**) 1 layer, (**b**) 2 layers, (**c**) 3 layers, (**d**) 4 layers.

Figure 10 shows cross-sectional images of TiB_2_ coatings with different numbers of layers prepared under 100 V–60 μF conditions. It can be seen that increasing the number of TiB_2_ layers leads to an increase in TiB_2_ thickness, but the thickness does not change proportionally. This is because during the deposition process, the high-speed rotating electrode cuts the coating, thereby reducing its thickness.

The primary crystal structures of TiB_2_ are lath-shaped crystals and large flake-shaped crystals. Based on crystal growth rates and mechanisms, Abdel et al. suggested that crystal morphology under non-equilibrium conditions depends on the attachment energy of crystal planes [36]. The crystal growth of TiB_2_ during the ESD process is under extreme non-equilibrium conditions; therefore, its crystal growth and morphology may be mainly controlled by the attachment energy of its crystal planes. Calculations by Zhao [37] and others indicate that the growth rates of TiB_2_ low-index planes are (0001) < (10$\bar{1}$0) < (10$\bar{1}$1) < (1$\bar{2}$10) < (1$\bar{2}$11). Therefore, the crystallographic characteristics of TiB_2_ also determine the anisotropy of its crystal growth, ultimately dictating the crystal morphology. When TiB_2_ nuclei reach a critical size, the (1$\bar{2}$10) and (1$\bar{2}$11) planes have higher growth rates, while the (0001), (10$\bar{1}$0) and (10$\bar{1}$1) planes have lower growth rates. When adjacent TiB_2_ crystals come into contact or when the solid/liquid interface front of TiB_2_ crystals on high-index planes fails to capture sufficient Ti and B atoms, the growth of TiB_2_ on high-index planes stagnates, while low-index planes continue to grow. This eventually causes TiB_2_ crystals to be enveloped by low-index planes such as (10$\bar{1}$0) and (10$\bar{1}$1), forming the flake-shaped crystals shown in Figure 10. Therefore, it may be concluded that the lath-shaped morphology of TiB_2_ is due to its faceted crystal growth characteristics, which is also reflected in XRD detection showing the highest diffraction intensity for the (101) plane of TiB_2_, followed by the (100) and (001) planes. Research by Higashi [38] and others shows that when TiB_2_ solidifies and crystallizes above 1000 °C, some Ti and B react to grow larger single crystals with good flake structures and large (0001) planes. This aligns with the large flake structures observed in our coatings.

### 3.7. Molten Aluminum Corrosion Performance of the TiB_2_ Coatings

Taking into account the microhardness, interdiffusion degree of substrate elements and tribological properties of the coatings, the TiB_2_ coating prepared at 60 μF exhibited the highest hardness, the least Fe element diffusion and a moderate and stable friction coefficient. Therefore, 60 μF was determined as the optimal deposition parameter and used for subsequent corrosion performance tests in this study.

**Figure 11:**
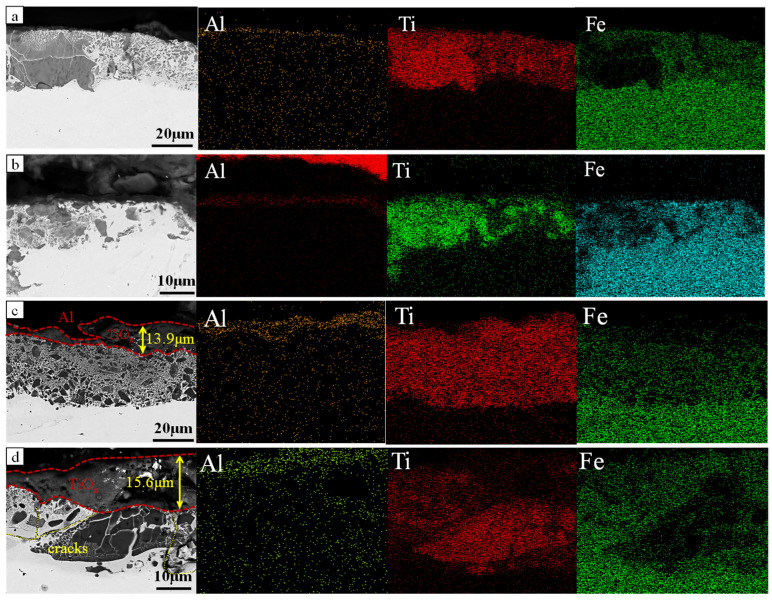
Cross-section morphology under different melt Al corrosion times: (**a**) 0.5 h, (**b**) 1 h, (**c**) 2 h, (**d**) 4 h.

Figure 11a,b show that after immersion in molten aluminum for 0.5 h and 1 h, the coating structure remains intact, and the molten aluminum did not penetrate the coating to corrode the substrate. As is clearly shown in Figure 11c, the coating was partially oxidized after 2 h of immersion in high-temperature molten aluminum, with a maximum oxide layer thickness of 13.9 μm. The underlying coating structure remained intact, maintained good adhesion to the substrate, and no obvious delamination was observed. The substrate was not eroded by the high-temperature aluminum melt. After the immersion time is increased to 4 h, the thickness of the surface oxide layer increases to ~15.6 μm. Cracks appear within the coating, both perpendicular and parallel to the surface, as shown in Figure 11d. The generation of cracks may be due to prolonged exposure to high temperatures, which intensifies elemental diffusion between the coating and the substrate. During cooling, differences in the thermal expansion coefficients of various components lead to the formation of thermal cracks.

**Figure 12:**
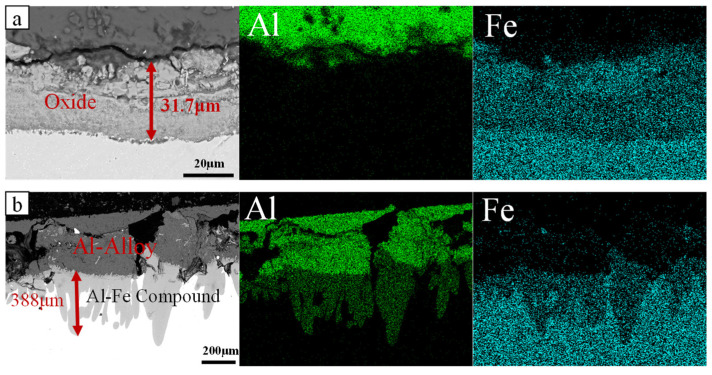
Cross-section morphology of as-built sample in melt Al for 4 h: (**a**) air contact area, (**b**) molten aluminum soaking area.

Figure 12 shows cross-sectional images of different regions of an as-built sample after immersion in high-temperature molten aluminum for 4 h. Figure 12a shows the area where the sample contacts both the high-temperature molten aluminum and air. A dense oxide layer approximately 31.7 μm thick forms on the sample surface, separating the substrate from the high-temperature aluminum melt and providing a certain degree of protection to the substrate. In the area completely covered by molten aluminum, jagged Fe-Al compounds penetrating into the substrate are observed, with a maximum depth of ~388 μm, causing severe corrosion to the substrate.

**Figure 13:**
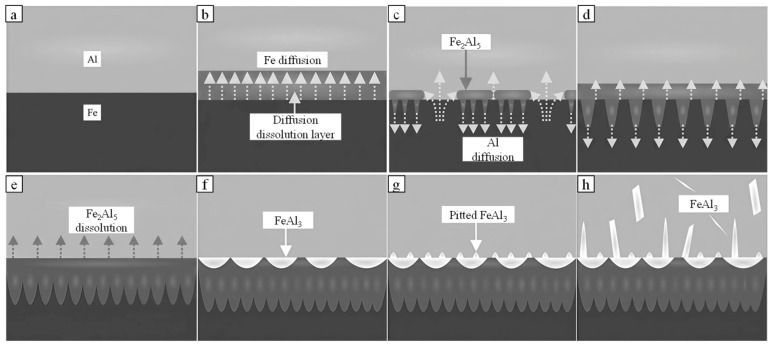
The growth of IMCs between liquid Al and Fe: (**a**) original interface, (**b**) Fe diffusion, (**c**) Fe_2_Al_5_ phase, (**d**) diffusion distance of Al becomes longer, (**e**) Fe_2_Al_5_ dissolves, (**f**) formation of FeAl_3_ phase, (**g**) pitted FeAl3, (**h**) needle-shaped FeAl_3_ phase.

Based on the experimental observations, we put forward the following speculations on the corrosion aging mechanism of iron-based materials immersed in high-temperature molten aluminum. The failure process of Fe-based materials in high-temperature aluminum melt can be roughly divided into three stages: wetting of the Fe-based material by the Al melt, dissolution of Fe by the Al melt, and reactive diffusion of Al into the Fe-based substrate. The reaction between liquid Al and solid Fe generates intermetallic compounds (IMCs) such as Fe_2_Al_5_ and FeAl_3_. The formation process of the IMCs is illustrated in Figure 13. In the initial stage of immersion, Fe atoms diffuse into the molten Al and form a diffusion layer (Figure 13a,b). Subsequently, chemical reactions occur at the interface between Al and Fe atoms, forming a continuous thin layer and tongue-like Fe_2_Al_5_ phase (Figure 13c,d). As the immersion time increases, the Fe_2_Al_5_ phase grows continuously into the substrate, which lengthens the diffusion distance of Al atoms and slows down the growth rate of the Fe_2_Al_5_ phase. Meanwhile, it begins to dissolve into the Al melt (Figure 13e). When the sample starts to cool, part of the Fe_2_Al_5_ phase at the corrosion interface transforms into the FeAl_3_ phase due to supersaturation of Al atoms (Figure 13f). After a period of cooling, pre-eutectic pitted and needle-shaped FeAl_3_ phases precipitate on the initial Fe_2_Al_5_ phase, followed by the formation of a eutectic FeAl_3_ phase in the melt (Figure 13g,h) [39].

## 4. Conclusions

In this work, the effects of heat treatments on microstructures and properties of a 18Ni350 maraging steel prepared by SLM and microstructure and corrosion resistance to the high-temperature molten aluminum of the TiB_2_ coatings deposited on the 18Ni350 surface via the ESD technique were preliminarily investigated. The following conclusions are drawn:

(1) Heat treatment significantly altered the microstructure morphology of SLM-fabricated 18Ni350 maraging steel. The as-built sample exhibited elongated laser track traces in the cross-section and a fish-scale-like morphology in the longitudinal section. After solution treatment, the microstructure was dominated by lath martensite. With increasing aging temperature, the cellular structure in the sample gradually deformed and discrete island-like structures began to emerge. The microstructure was fully homogenized after combined solution-aging treatment.
(2) Heat treatment markedly modified the mechanical properties of the samples. In the as-built state, the sample had a microhardness of 341.3 HV_0.1_, an ultimate tensile strength (UTS) of 1122 MPa, a yield strength (YS) of 862 MPa, and an elongation (EL) of 12.4%. The sample achieved the optimal comprehensive mechanical properties after solution treatment at 800 °C for 1 h followed by aging at 520 °C for 6 h, with a microhardness of 711.5 HV_0.1_, a UTS of 2476 MPa, a YS of 2408 MPa, and an EL of 4.6%.
(3) The TiB_2_ coating deposited by ESD exhibited the best deposition quality at 100 V and 60 μF. No obvious material heterogeneity was observed in the coating, which demonstrated good stability in high-temperature molten aluminum. Partial oxidation occurred after 4 h of immersion, while the substrate remained uncorroded by the molten aluminum.

In this study, high-performance 18Ni350 maraging steel was successfully prepared, and the effects of different heat treatment processes on its microstructure and mechanical properties were preliminarily investigated. However, due to limitations in experimental time and conditions, further research is still required. Only a limited discussion has been conducted on the failure behavior of 18Ni350 steel in high-temperature molten aluminum.

## Figures and Tables

**Table 1:** Chemical composition of 18Ni 350 maraging steel powder (wt%).

Ni	Co	Mo	Ti	Al	S	C	Si	Mn	P	Fe
18.04	12.01	4.40	1.24	0.073	≤0.003	0.011	0.015	0.012	0.0060	Bal

**Table 2:** Different forming process parameters of SLM.

Specimens	d/μm	v/mm·s^−1^	h/μm	P/W	E/J·mm^−3^
1	30	1000	100	300	100
2	30	1000	100	350	117
3	30	1000	80	300	125
4	30	500	100	350	233
5	30	500	100	300	200
6	30	500	80	350	292
7	50	1000	100	300	60
8	50	1000	100	350	70
9	50	1000	80	300	75
10	50	500	100	350	140
11	50	500	100	300	120
12	50	500	80	350	175

**Table 3:** Heat treatment processes and corresponding sample names.

Heat Treatments	Sample Names
/	No heat treatment after fabrication (as built)
Solution Treatment	800 °C-1 h-Water quench (ST 800) 850 °C-1 h-Water quench (ST 850)
Aging Treatment	500 °C-6 h-Air cooling (DA 500) 520 °C-6 h-Air cooling (DA 520) 540 °C-6 h-Air cooling (DA 540)
Solution-Aging Treatment	800 °C-1 h-Water quench + 500 °C-6 h-Air cooling (SAT 800-500) 800 °C-1 h-Water quench + 520 °C-6 h-Air cooling (SAT 800-520) 800 °C-1 h-Water quench + 540 °C-6 h-Air cooling (SAT 800-540) 850 °C-1 h-Water quench + 520 °C-6 h-Air cooling (SAT 850-520)

**Table 4:** Tensile properties in different heat treatment conditions.

Specimens	UTS (MPa)	YS (MPa)	EL (%)
As Built	1122 ± 3	862 ± 7	12.4 ± 0.2
ST 800	1140 ± 3	877 ± 9	11.7 ± 0.4
ST 850	1133 ± 1	881 ± 5	14.9 ± 0.8
DA 500	2314 ± 10	2238 ± 23	4.7 ± 0.2
DA 520	2325 ± 4	2276 ± 23	5.0 ± 0.5
DA 540	2231 ± 7	2157 ± 7	6.8 ± 0.6
SAT 800-500	2471 ± 8	2414 ± 25	2.7 ± 0.2
SAT 800-520	2476 ± 3	2408 ± 6	4.6 ± 0.3
SAT 800-540	2390 ± 6	2312 ± 5	4.8 ± 0.1
SAT 850-520	2492 ± 6	2289 ± 3	3.9 ± 0.2

## Data Availability

The original contributions presented in this study are included in the article. Further inquiries can be directed to the corresponding author.
